# The Role of Gut Microbiota in Insomnia: A Systematic Review of Case–Control Studies

**DOI:** 10.3390/life15071086

**Published:** 2025-07-10

**Authors:** Yun Wang, Suyi Xie, Sizhe Chen, Chenyu Li, Yeuk Lam Chan, Ngan Yin Chan, Yun Kwok Wing, Francis K. L. Chan, Qi Su, Siew C. Ng

**Affiliations:** 1 Microbiota I-Center (MagIC), Hong Kong SAR, China; 1155202358@link.cuhk.edu.hk (Y.W.); 1155202847@link.cuhk.edu.hk (S.C.); chenyuli@magic-inno.hk (C.L.); ashleyychan@cuhk.edu.hk (Y.L.C.); fklchan@cuhk.edu.hk (F.K.L.C.); 2 Department of Medicine and Therapeutics, The Chinese University of Hong Kong, Hong Kong SAR, China; 3 Li Chiu Kong Family Sleep Assessment Unit, Department of Psychiatry, The Chinese University of Hong Kong, Hong Kong SAR, China; suyixie@cuhk.edu.hk (S.X.); rachel.chan@cuhk.edu.hk (N.Y.C.); ykwing@cuhk.edu.hk (Y.K.W.); 4 Li Ka Shing Institute of Health Sciences, Faculty of Medicine, The Chinese University of Hong Kong, Hong Kong SAR, China; 5 Centre for Gut Microbiota Research, The Chinese University of Hong Kong, Hong Kong SAR, China; 6 New Cornerstone Science Laboratory, The Chinese University of Hong Kong, Hong Kong SAR, China

**Keywords:** gut microbiota, insomnia, sleep, comparison, systematic review

## Abstract

Background: Insomnia is one of the most prevalent health concerns and has a major impact on human health and quality of life. Increasing evidence indicates the gut microbiota’s role in sleep regulation through the gut–brain axis. This systematic review aims to summarise current evidence on the role of gut microbiota alterations in insomnia. Methods: We searched PubMed, Embase, and Cochrane Library through November 2024 for case–control studies comparing gut microbiota in insomnia subjects and controls. The primary outcome was changes in microbiota diversity and bacteria taxonomy. Results: We included 15 case–control studies from 14 articles, consisting of 1321 subjects (603 insomnia; 718 controls). Eight studies showed reduced alpha diversity and eleven showed altered beta diversity in insomnia subjects. Depletions of specific taxa such as Lactobacillales (class Bacilli), *Faecalibacterium*, and *Lachnospira* and the enrichment of Actinobacteria, Bacteroidales (class Bacteroidia), and several genera, including *Streptococcus*, *Blautia*, *Lactobacillus*, *Clostridium*, *Holdemanella*, and *Eubacterium hallii*, were observed in insomnia subjects. There was a negative association between insomnia severity and abundance of *Faecalibacterium* and *Lachnospira*, and positive associations with *Blautia*. Conclusions: This systematic review identifies specific alterations in gut microbiota among insomnia subjects characterised by taxonomic changes that may serve as promising therapeutic targets for sleep disorders.

## 1. Introduction

Insomnia is a common clinical condition characterised by difficulties in falling asleep, staying asleep, or experiencing non-restorative sleep [1]. It affects 10–20% of the adult population worldwide, with substantial impacts on physical health, mental well-being, and overall quality of life [1]. The COVID-19 pandemic has further aggravated sleep problems, with a reported global prevalence of sleep disturbances (including poor sleep quality and insomnia) reaching 40% [2]. Around 50% of cases will develop into a chronic course [1], which poses particularly substantial risks for the development of cardiovascular and mental disorders, such as major depressive disorder and cognitive deficits [3,4,5].

Recent advances in neuroscience and microbiology have highlighted the intricate connections between the gut and the brain, collectively known as the gut–brain axis [6]. The gut microbiota, a complex community of microorganisms residing in the gastrointestinal tract, affects brain function and human behaviour through the production of metabolites, modulation of the immune system, and interaction with the central nervous system, and thereby plays an important role in various mental disorders [7,8,9]. Several studies have also shown the associations between altered gut microbiota and insomnia [10,11,12]. Furthermore, microbiome-targeted interventions, including probiotics, prebiotics, synbiotics, postbiotics, dietary interventions, and faecal microbiota transplantation, have demonstrated potential in alleviating insomnia symptoms and improving sleep quality [9,13]. However, the causal relationship between gut microbiota and insomnia is still unclear [12,14,15]. Some studies suggest that certain microbial taxa may be either depleted or enriched in individuals with insomnia, potentially playing a role in the disorder’s pathophysiology [14,15], while other studies have reported conflicting findings [16,17], highlighting the necessity for a thorough synthesis of the available evidence.

This systematic review aims to fill this gap by rigorously evaluating and synthesising existing research on the relationship between the gut microbiota and insomnia. By analysing community-level measures of gut microbiota composition and taxonomic findings across different levels, the review seeks to identify consistent patterns and potential biomarkers linked to insomnia. Furthermore, it aims to explore the implications of these findings for understanding the pathogenesis of insomnia and for the development of innovative therapeutic strategies that target the gut microbiota.

## 2. Materials and Methods

### 2.1. Search Strategy

The reporting of this systematic review is based on the Preferred Reporting Items for Systematic Reviews and Meta-Analyses (PRISMA) Statement 2020 [18]. We performed a systematic search of PubMed, Embase, and Cochrane Library from inception to November 2024 to identify case–control studies comparing gut microbiota in subjects with insomnia and controls with normal sleep patterns.

The search strategy involved crossmatching keywords selected based on key terms and the PubMed Medical Subjects Headings (MeSH). The Boolean logic operators of (OR, AND) were used to develop the search in an [All Fields] search. Each database’s advanced search characteristics were used to change the search syntax. In the search, the following keywords were used: “insomnia” OR “sleep disorder*” OR “sleep disturbance*” OR “sleep problem*” OR “sleep difficult*” OR “sleep initiation” OR “sleep maintenance” OR “early awakening” OR “sleep quality” OR “poor sleep” OR “sleeplessness” AND “gut microbiota” OR “gut microbiome” OR “intestinal microbiota” OR “intestinal microbiome” OR “gastrointestinal microbiota” OR “gastrointestinal microbiome” OR “gut flora” OR “gut bacteria” OR “intestinal flora” OR “intestinal bacteria”.

### 2.2. Study Selection

The inclusion criteria were as follows: (1) studies which applied an observational case–control design, (2) performed gut microbiota analysis and reported diversity or abundance measures, and (3) sampled a population with insomnia disorder or insomnia symptoms. Studies were excluded if they did not provide the microbiome data, were not in English, or were only available as conference proceeding abstracts.

### 2.3. Data Extraction

Information was extracted using a predesigned template by two authors and cross-checked. We extracted publication details, participant demographic and clinical characteristics, and methodological information. As primary outcomes of interest, we extracted community-level measures of gut microbiota composition (alpha and beta diversity) and taxonomic findings at the phylum, class, order, family, genus, and species levels (relative abundance). Control samples were defined as individuals without insomnia. The secondary outcome was the correlation between insomnia severity and microbiota alterations.

### 2.4. Quality Assessment

The Newcastle–Ottawa scale (NOS) containing three criteria (selection, comparability, and exposure) was used to assess the quality of the included case–control studies, following the standard 9-point scale. No studies were excluded owing to quality concerns.

### 2.5. Qualitative Synthesis

For the relative abundance of microbial taxa, we summarised the findings for each taxon in each study and labelled these as increased, decreased, or of no significance compared to the controls.

## 3. Results

### 3.1. Study Selection and Literature Flow

**Figure 1:**
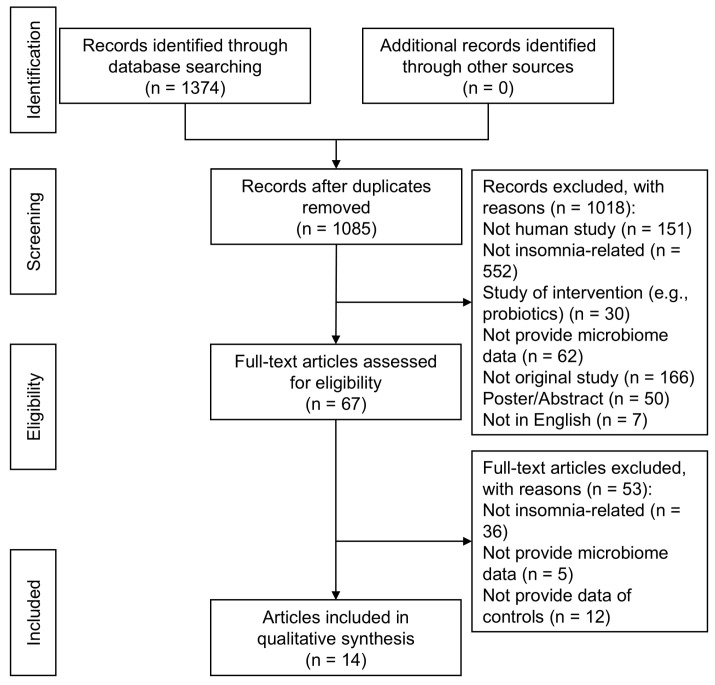
PRISMA flowchart.

Initially, 1374 citations were retrieved. After screening titles and abstracts for relevance and removing duplicates, 1307 articles were excluded. The remaining 67 articles underwent a full-text review. Upon further examination, an additional 53 articles were excluded for not meeting the inclusion criteria. Consequently, this process yielded 14 articles suitable for the final analysis (Figure 1).

### 3.2. Study Characteristics and Population Demographics

The analysis included 15 studies from 14 articles [12,14,15,16,17,19,20,21,22,23,24,25,26,27], consisting of 603 insomnia patients and 718 controls (Table 1). The majority of studies (12, 80.0%) were conducted in China, with the remaining studies distributed across Japan (1, 6.7%) [23], Italy (1, 6.7%) [19], and Russia (1, 6.7%) [15]. Adult populations were the focus in 13 articles across 14 studies [12,14,15,17,19,20,21,22,23,24,25,26,27], while only one examined paediatric subjects [16]. Six studies included participants with comorbid conditions [16,17,21,22,23,25]. Five studies from four articles [14,21,24,27] employed the Diagnostic and Statistical Manual of Mental Disorders, Fifth Edition (DSM-5) criteria, while three utilised the International Classification of Sleep Disorders, Third Edition (ICSD-3) [12,15,19] to diagnose insomnia disorders. Additionally, six studies used the Pittsburgh Sleep Quality Index (PSQI) to assess insomnia symptoms [17,20,22,23,25,26], and one study employed the Children’s Sleep Habits Questionnaire (CSHQ) to evaluate insomnia symptoms in children [16].

Five articles specified the insomnia subtype, with four focusing on chronic insomnia disorder defined as having insomnia longer than 3 months based on DSM-5 or ICSD-3 [12,15,19,24] and one including both acute (having insomnia longer than 1 week but shorter than 3 months) and chronic cases based on DSM-5 [14]. For microbiome assessment, 16S rRNA sequencing was utilised in all 15 studies, but there were differences in the variable region sequenced. Two studies used V1-V2 [23,24], ten studies from nine articles sequenced V3-V4 [12,14,16,19,20,21,22,26,27], and three studies did not specify the gene region [15,17,25].

### 3.3. Microbial Diversity Patterns in Insomnia

**Figure 2:**
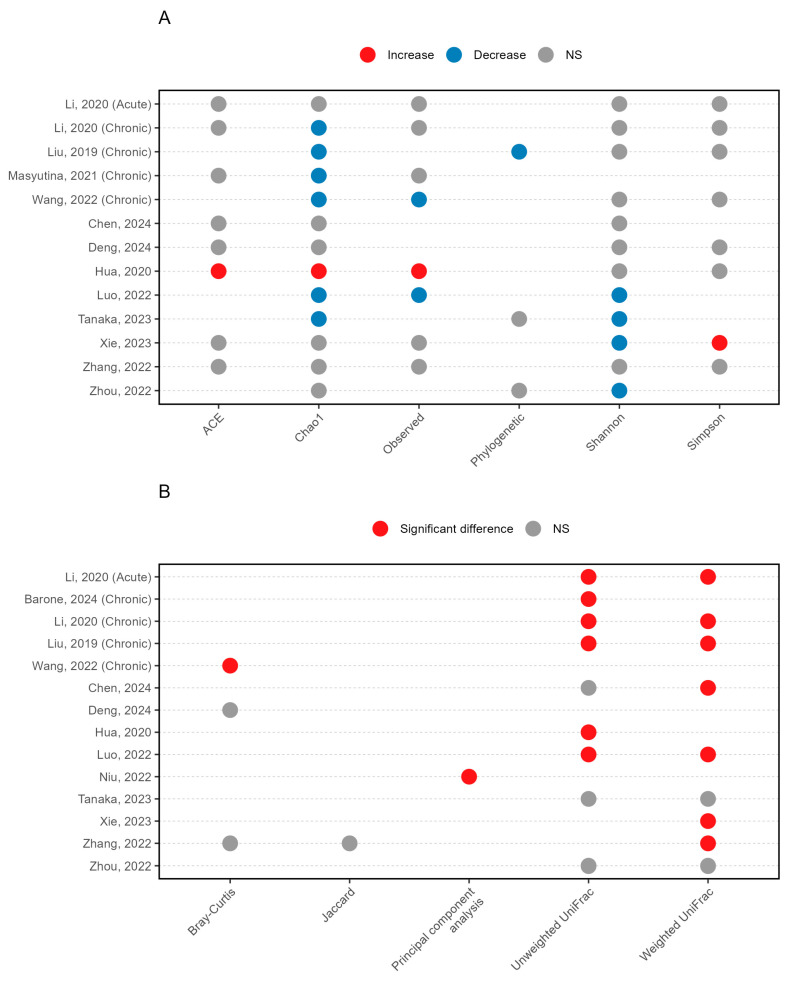
Differences in alpha (**A**) [12,14,15,16,17,20,21,22,23,24,26,27] and beta (**B**) [12,14,16,17,19,20,21,22,23,24,25,26,27] diversity indices between insomnia subjects and controls across studies. ACE, abundance-based coverage estimator. NS, no significance.

Among the 14 studies reporting alpha diversity, 13 studies from 12 articles specified their diversity indices [12,14,15,16,17,20,21,22,23,24,26,27], while 1 study [19] was excluded from analysis due to unspecified metrics. Six indices were employed to assess alpha diversity, including estimates of richness (observed, Chao1, and abundance-based coverage estimator), richness/evenness (Shannon, Simpson), and biodiversity (phylogenetic diversity). The most widely used indices were Chao1 (13/13) and Shannon (12/13) (Figure 2A). Overall, the majority of investigations (8/13) revealed decreased gut microbiota diversity in subjects with insomnia when compared to controls [12,14,15,17,22,23,24,27].

Beta diversity was reported in 13 articles across 14 studies (Figure 2B), while most studies utilised weighted (9/14) [12,14,17,20,22,23,26,27] or unweighted uniFrac distances (9/14) [12,14,16,19,20,22,23,27] to compare beta diversity between insomnia patients and controls. Notably, eleven studies revealed consistent differences in beta diversity between subjects with insomnia and controls in ten articles [12,14,16,17,19,20,22,24,25,26].

Overall, these findings confirmed the reduced diversity and distinct composition pattern of the gut microbiome in subjects with insomnia.

### 3.4. Taxonomic Alterations in Insomnia

**Figure 3:**
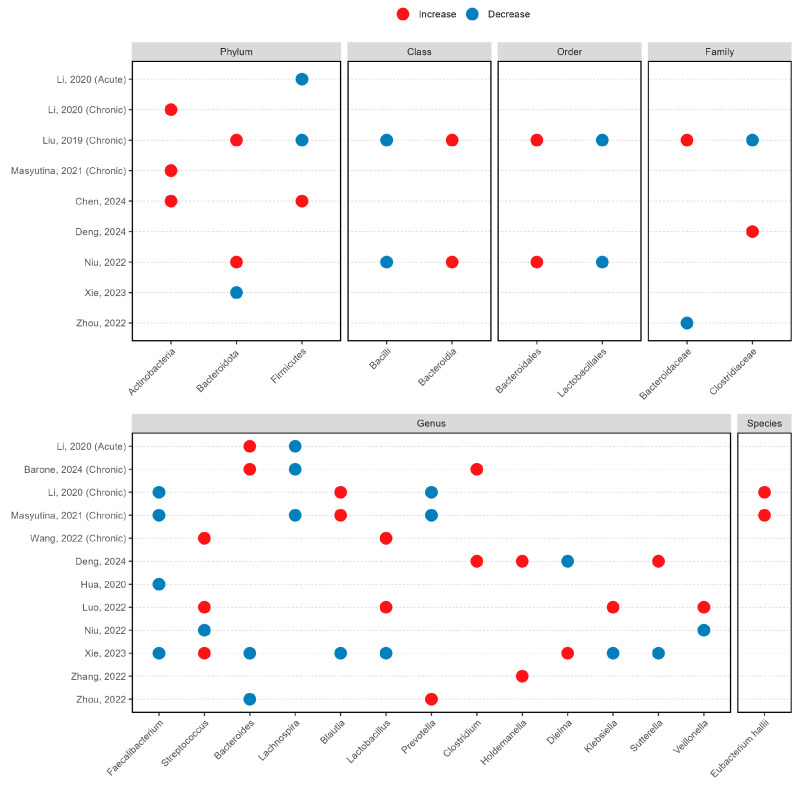
Differences in relative abundance of microbial taxa in insomnia subjects compared to controls reported by at least 2 studies [12,14,15,16,17,19,20,21,22,24,25,26,27].

At the phylum level, elevated Actinobacteria abundance was observed in subjects with insomnia across three studies [14,15,20] (Figure 3). The evidence for Bacteroidota [12,17,25] and Firmicutes [12,14,20] was inconsistent, and data were insufficient for comprehensive analysis of Patescibacteria [17] and Campilobacterota [17] (Figure S1). At the order level, two studies [12,25] reported significant enrichment of Bacteroidales (class Bacteroidia) and reductions in Lactobacillales (class Bacilli) among insomnia patients (Figure 3). Family-level analyses also yielded heterogeneous results, with contradictory findings for Bacteroidaceae [12,27] and Clostridiaceae [12,21] across different studies (Figure 3).

At the genus level, four studies reported significant decreases in *Faecalibacterium* [14,15,16,17], and *Lachnospira* exhibited depletion in two chronic insomnia cohorts [15,19] and one acute insomnia cohort [14] (Figure 3). *Bacteroides* demonstrated variable patterns between subjects with and without insomnia, with increases in two studies [14,19] and decreases in two studies [17,27] (Figure 3). *Streptococcus* was enriched in subjects with insomnia in three studies [17,22,24] but depleted in one [25]. *Blautia* [14,15,17], *Lactobacillus* [17,22,24] and *Prevotella* [14,15,27] showed conflicting associations with insomnia, while *Clostridium* [19,21] and *Holdemanella* [21,26] demonstrated consistent enrichment in subjects with insomnia (Figure 3). Notably, *Eubacterium* enrichment was reported by Luo et al. [22] (Figure S1), with *Eubacterium hallii* being the only species showing a consensus on enrichment across two studies [14,15] (Figure 3).

### 3.5. Microbiota-Insomnia Severity Associations

**Figure 4:**
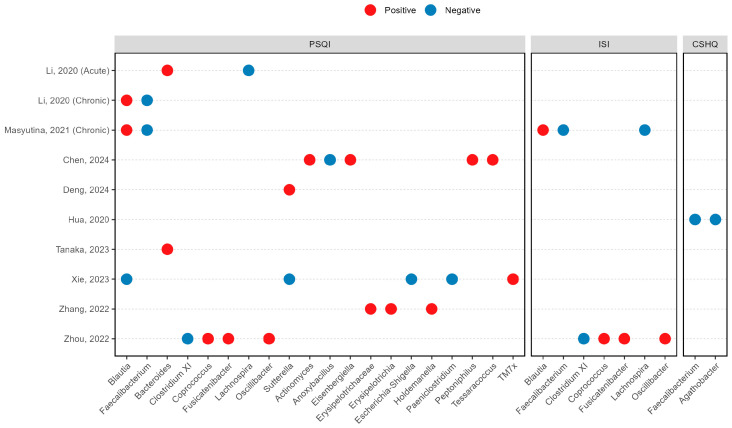
Significant associations between microbial taxon and insomnia severity [14,15,16,17,20,21,23,26,27]. CSHQ, Children Sleep Habits Questionnaire; ISI, Insomnia Severity Index; PSQI, Pittsburgh Sleep Quality Index.

Correlations between microbial taxa and sleep measures were analysed using Spearman correlation in multiple cohorts: nine studies used the Pittsburgh Sleep Quality Index (PSQI) in eight articles [14,15,17,20,21,23,26,27], two used the Insomnia Severity Index (ISI) [15,27], and one used the Children’s Sleep Habits Questionnaire (CSHQ) in paediatric subjects [16] (Figure 4). The genus *Faecalibacterium* demonstrated significant negative correlations with PSQI scores in two independent chronic insomnia cohorts [14,15] (Figure 4). This negative association was further supported by negative correlations with ISI scores [15] and CHSQ scores [16]. In addition, the genus *Lachnospira* also exhibited negative correlations with insomnia severity in both acute [14] and chronic [15] insomnia populations. On the other hand, both *Blautia* [14,15] and *Bacteroides* [14,23] showed positive correlations with insomnia severity in two cohorts. Contradictory findings emerged for the genus *Sutterella*, with one study reporting positive correlations [21] and another showing negative associations [17] with insomnia severity measures.

### 3.6. Microbiome Signatures in Chronic Insomnia Disorders

Among the included studies, five specifically investigated chronic insomnia disorders [12,14,15,19,24] (Table 1), yielding consistent patterns in microbial diversity analyses. Four studies demonstrated decreased alpha diversity as measured by Chao1 index [12,14,15,24], and significant alterations in beta diversity were reported in four studies [12,14,19,24] (Figure 2). Taxonomic analyses revealed enrichment of phylum Actinobacteria in two chronic insomnia cohorts [14,15] (Figure 3). At the genus level, consistent patterns emerged with depletion of *Faecalibacterium* [14,15], *Lachnospira* [15,19] and *Prevotella* [14,15] alongside enrichment of *Blautia* [14,15] across studies. At the species level, *Eubacterium hallii* was enriched in two chronic insomnia cohorts [14,15].

### 3.7. Quality of Evidence

The methodological quality of the included studies, assessed using NOS, yielded scores ranging from 4 to 7 (Table 2). All 15 studies clearly defined insomnia diagnostic criteria and selection methods. Control group documentation was complete in five studies (33.3%), while nine studies (60.0%) demonstrated matched age/sex distributions between cases and controls through statistical comparisons. Microbiologist blinding during laboratory analysis was unreported in all studies, though all reported equivalent sample attrition rates between groups for microbiota analysis.

## 4. Discussion

This systematic review analyses the role of gut microbiota alterations in insomnia. Our analysis revealed reduced diversity, distinct composition patterns, and taxonomic markers in the gut microbiota in subjects with insomnia, highlighting the potential of targeted gut microbiome modulation to aid the treatment of insomnia.

A key finding is the enrichment of phylum Actinobacteria in insomnia patients. Previous research has demonstrated an increase in Actinobacteria in patients with Parkinson’s disease [28]. Notably, Actinobacteria showed positive correlations with inflammatory markers, including neutrophil count and monocyte count/percentage. Furthermore, another study has suggested that Actinobacteria may influence neurotransmitter-associated metabolites, particularly tryptophan metabolism [29], which is crucial for sleep–wake regulation [30]. These findings collectively suggest that Actinobacteria may influence sleep regulation through multiple mechanisms, including inflammatory pathways and neurotransmitter metabolism along the gut–brain axis.

At the genus level, *Faecalibacterium* was depleted in subjects with insomnia and exhibited negative correlations with insomnia severity. As a well-known anti-inflammatory bacteria [31], *Faecalibacterium* exerts its beneficial effects primarily through the production of butyrate [32,33], a short-chain fatty acid crucial for maintaining mucosal integrity and modulating inflammation by suppressing pro-inflammatory cytokines and promoting anti-inflammatory mediators [34,35,36]. The relationship between *Faecalibacterium* and sleep is bidirectional: while sleep deprivation (SD) impairs intestinal barrier function and reduces *Faecalibacterium* abundance [37], an experimental study has shown that *Faecalibacterium prausnitzii* pretreatment can increase faecal butyrate levels and mitigate SD-induced gut damage in mice [38]. The depletion of *Faecalibacterium* in insomnia patients mirrors findings from several immune-mediated inflammatory diseases [39,40], suggesting a shift toward a pro-inflammatory gut environment. Clinical evidence further supports its role in sleep regulation, as higher *Faecalibacterium* abundance has been significantly associated with improved sleep quality scores in patients with bipolar disorder [41]. These findings indicate that therapeutic strategies targeting *Faecalibacterium* restoration could potentially improve sleep quality by enhancing intestinal barrier function and maintaining gut microbiota homeostasis.

Insomnia is frequently co-morbid with depression, with a bidirectional relationship between these two disorders [4,42]. Individuals with insomnia are five times as likely to present with anxiety or depression compared to individuals without insomnia [43]. The depletion of *Lachnospira* observed in this review aligns with existing evidence linking reduced *Lachnospira* abundance to various neuropsychiatric conditions, including anxiety and depression [44,45]. The presence of *Eubacterium hallii*, known for its role in butyrate production [46] and potential to modify metabolism [47], in higher abundance in insomnia patients is intriguing and warrants further investigation to understand its impact on sleep physiology. The heterogeneous findings for certain taxa, particularly *Bacteroides* [48], *Lactobacillus* [49], *Prevotella* [50], and *Blautia* [51], highlight the complexity of microbiota–sleep interactions and suggest potential confounding factors.

The methodological assessment using NOS revealed important limitations in current evidence. While insomnia patient criteria were well-defined, control group documentation was often inadequate. Issues including incomplete demographic matching and a lack of blinded microbiological analysis highlight the need for more rigorous study designs. These limitations may introduce selection and detection biases, particularly for taxa with small effect sizes. While 16S rRNA sequencing was predominantly used, methodological heterogeneity in sampling, processing, and analysis may contribute to some inconsistent findings across studies. Additionally, 16S rRNA sequencing limits a deeper understanding of the mechanisms involved, indicating the need for metagenomic sequencing. The limited investigation of insomnia subtypes (6/15 studies) represents a significant knowledge gap in understanding subtype-specific microbiota patterns. Importantly, the lack of reported quantitative data on microbial diversity and relative abundance precluded meaningful meta-analytical visualisation through forest plots, significantly limiting our ability to perform comparative statistical analyses across studies. These collective limitations emphasise the need for standardised protocols and comprehensive data reporting in future research.

## 5. Conclusions

In conclusion, this review identifies specific gut microbiota alterations in insomnia, particularly regarding community structure and specific taxa. These findings advance our understanding of the gut–brain axis in sleep regulation and suggest potential therapeutic targets. However, more rigorous, standardised studies are needed to strengthen these associations and develop effective microbiota-based interventions.

To advance this field, future studies should employ larger sample sizes and standardised methodologies to minimise heterogeneity in microbiota analysis and insomnia assessment. Mechanistic investigations exploring microbial metabolites and their effects on sleep-related pathways are needed to establish causality. Additionally, clinical trials evaluating microbiota-targeted interventions, such as probiotics, prebiotics, or dietary modifications, could pave the way for novel insomnia treatments. Addressing these gaps will be crucial for translating gut microbiota research into clinically actionable strategies for sleep disorder management.

## Figures and Tables

**Table 1:** Characteristics of included studies.

Study	Country	Comorbid Diseases	Diagnosis Criteria for Insomnia	Age Group	Subtype	No. of Insomnia Cases	No. of Control Cases	Type of Specimen	DNA Extraction Method	Microbiome Assessment Method
Barone, 2024 [19]	Italy	NR	ICSD-3	Adult	Chronic	54 (F)	42 (F)	Stool	QIAamp DNA Stool Mini Kit (QIAGEN, Hilden, Germany)	16S rRNA V3-V4
Chen, 2024 [20]	China	NR	PSQI ≥ 5	Adult	NR	26 (M) 65 (F)	42 (M) 105 (F)	Stool	MGIEasy fecal genomic DNA (meta) extraction kit (BGI, Shenzhen, China)	16S rRNA V3-V4
Deng, 2024 [21]	China	Methamphetamine users during abstinence	DSM-5 PSQI ≥ 7	Adult	NR	14 (M) 7 (F)	35 (M) 14 (F)	Stool	DNA extraction kit (MN^®^ NucleoSpin 96 Soi kit, Düren, Germany)	16S rRNA V3-V4
Hua, 2020 [16]	China	Autism	CSHQ ≥ 41	Child	NR	48 (M) 12 (F)	52 (M) 8 (F)	Stool	OMEGA DNA Kit (Omega Bio-Tek, Norcross, GA, USA)	16S rRNA V3-V4
Li, 2020 [14]	China	NR	DSM-5	Adult	Acute	5 (M) 15 (F)	20 (M) 18 (F)	Stool	HiPure Stool DNA Kits B (D3141-03B, Guangzhou meiji biotechnology Co., Ltd., Guangzhou, China)	16S rRNA V3-V4
Li, 2020 [14]	China	NR	DSM-5	Adult	Chronic	13 (M) 25 (F)	20 (M) 18 (F)	Stool	HiPure Stool DNA Kits B (D3141-03B, Guangzhou meiji biotechnology Co., Ltd., Guangzhou, China)	16S rRNA V3-V4
Liu, 2019 [12]	China	NR	ICSD-3	Adult	Chronic	10	10	Stool	ZR Fecal DNA Kit (Zymo Research, Irvine, CA, United States)	16S rRNA V3-V4
Luo, 2022 [22]	China	Minimal hepatic encephalopathy	PSQI > 5	Adult	NR	37 (M) 28 (F)	45 (M) 33 (F)	Stool	QIAamp Fast DNA Stool Mini Kit (Qiagen, Germantown, MD, USA)	16S rRNA V3-V4
Masyutina, 2021 [15]	Russia	NR	ICSD-3	Adult	Chronic	23 (M) 32 (F)	16 (M) 34 (F)	Stool	Means of phenol extraction	16S rRNA
Tanaka, 2023 [23]	Japan	Depression and anxiety	PSQI ≥ 9	Adult	NR	7 (M) 13 (F)	10 (M) 10 (F)	Stool	In-house method	16S rRNA V1-V2
Wang, 2022 [24]	China	NR	DSM-5	Adult	Chronic	13 (M) 27 (F)	10 (M) 30 (F)	Stool	Modified cetyl trimethyl-ammonium bromide (CTAB) methods	16S rRNA V1-V2
Xie, 2023 [17]	China	Ischemic stroke	PSQI > 5	Adult	NR	46 (M) 28 (F)	92 (M) 39 (F)	Stool	E.Z.N.A.^®^ soil DNA Kit (Omega Bio-Tek, Norcross, GA, USA)	16S rRNA
Niu, 2022 [25]	China	Traumatic brain injury	PSQI	Adult	NR	11 (M) 3 (F)	11 (M) 3 (F)	Stool	NR	16S rRNA
Zhang, 2022 [26]	China	NR	PSQI > 7	Adult	NR	10 (M) 7 (F)	7 (M) 10 (F)	Stool	QIAamp DNA stool Mini Kit (Qiagen, Hilden, Germany)	16S rRNA V3-V4
Zhou, 2022 [27]	China	NR	DSM-5 PSQI ≥ 11	Adult	NR	24	22	Stool	QIAamp^®^ Fast DNA stool mini kit (Qiagen, Hilden, Germany)	16S rRNA V3-V4

CSHQ, Children Sleep Habits Questionnaire; DSM-5, Diagnostic and Statistical Manual of Mental Disorders, Fifth Edition; ICSD-3, International Classification of Sleep Disorders, Third Edition; ISI, Insomnia Severity Index; NR, not recorded; PSQI, Pittsburgh Sleep Quality Index.

**Table 2:** Quality of each included study by the Newcastle–Ottawa scale.

	Selection				Comparability		Outcome		
Study	Is the Case Definition Adequate?	Representativeness of the Cases	Selection of Controls	Definition of Controls	Comparability of Baseline Characteristic 1 (Gender)	Comparability of Baseline Characteristic 2 (Age)	Ascertainment of Exposure	Same Method of Ascertainment for Cases and Controls	Non-Response Rate
Barone, 2024 [19]	1	1	1	1	1	0	0	0	1
Chen, 2024 [20]	1	1	0	1	1	1	0	0	1
Deng, 2024 [21]	1	1	1	1	1	1	0	0	1
Hua, 2020 [16]	1	1	0	1	1	1	0	0	1
Li, 2020 [14]	1	1	1	1	1	0	0	0	1
Li, 2020 [14]	1	1	1	1	1	0	0	0	1
Liu, 2019 [12]	1	1	0	1	0	0	0	0	1
Luo, 2022 [22]	1	1	0	1	1	1	0	0	1
Masyutina, 2021 [15]	1	1	0	1	1	1	0	0	1
Tanaka, 2023 [23]	1	1	0	1	1	1	0	0	1
Wang, 2022 [24]	1	1	0	1	0	0	0	0	1
Xie, 2023 [17]	1	1	0	1	1	1	0	0	1
Niu, 2022 [25]	1	1	0	1	1	1	0	0	1
Zhang, 2022 [26]	1	1	1	1	1	1	0	0	1
Zhou, 2022 [27]	1	1	0	1	1	0	0	0	1

## Data Availability

The data that support the findings of this study are available from the corresponding author, Q.S., upon reasonable request.

## Supplementary Materials

The following supporting information can be downloaded at: https://www.mdpi.com/article/10.3390/life15071086/s1, Figure S1: Differences in relative abundance of microbial taxa in insomnia disorder compared to controls reported by one study.
