# Supplementary material for: The Role of Gut Microbiota in Insomnia: A Systematic Review of Case–Control Studies
Source: Life (Basel). 2025 Jul 10;15(7):1086. doi: 10.3390/life15071086 (PMC12299568; doi:10.3390/life15071086)

Figure S1. Differences in relative abundance of microbial taxa in insomnia disorder compared to controls reported by one study. NS, no significance.

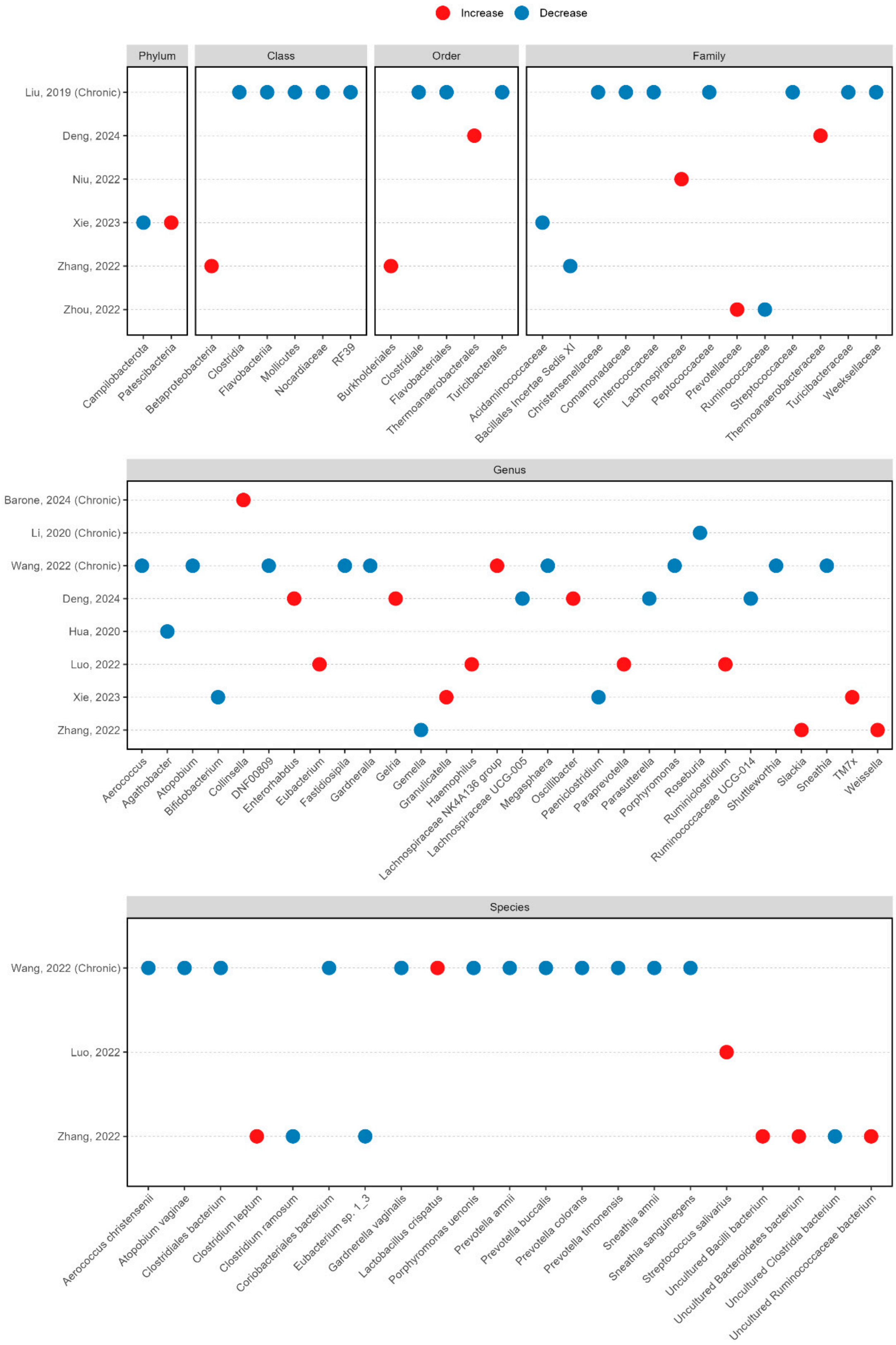

Supplement: Supplementary file 1 [file life-15-01086-s001.zip › life-3672617-supplementary.pdf]
